# Lipid-laden partially-activated plasmacytoid and CD4^−^CD8α^+^ dendritic cells accumulate in tissues in elderly mice

**DOI:** 10.1186/1742-4933-11-11

**Published:** 2014-07-29

**Authors:** Joanne K Gardner, Cyril DS Mamotte, Terrence McGonigle, Danielle E Dye, Connie Jackaman, Delia J Nelson

**Affiliations:** 1 Immunology and Cancer Group, School of Biomedical Sciences, Curtin University, Perth 6102, Western Australia, Australia; 2 School of Biomedical Sciences, CHIRI Biosciences Research Precinct, Curtin University, Perth 6102, Western Australia, Australia; 3 Curtin University of Technology, School of Biomedical Sciences, Bentley, Perth 6102 Western Australia, Australia

**Keywords:** Dendritic cell, Immunosenescence, Lipid uptake, Aged mice

## Abstract

**Background:**

Aging is associated with a decline in lymphocyte function however, little is known about dendritic cell (DC) subsets and aging. Aging is also associated with increasing circulating lipid levels and intracellular lipid accumulation modulates DC function. Whether age-associated increases in lipid levels influence DC biology is unknown. Thus, the effects of aging on DC subsets were assessed in vivo using young adult and elderly C57BL/6 J mice.

**Results:**

Major age-related changes included increased CD11c^+^ DC numbers in lymph nodes, spleens and livers, but not lungs, and significantly increased proportions of plasmacytoid (pDC) and CD4^-^CD8α^+^ DCs in lymph nodes and livers. Other changes included altered pDC activation status (decreased CD40, increased MHC class-I and MHC class-II), increased lipid content in pDCs and CD4^-^CD8α^+^ DCs, and increased expression of key mediators of lipid uptake including lipoprotein lipase, scavenger receptors (CD36, CD68 and LRP-1) in most tissues.

**Conclusions:**

Aging is associated with organ-specific numerical changes in DC subsets, and DC activation status, and increased lipid content in pDCs and CD4^-^CD8α^+^ DCs. Up-regulation of lipoprotein lipase and scavenger receptors by lipid-rich pDCs and CD4^-^CD8α^+^ DCs suggests these molecules contribute to DC lipid accumulation in the elderly. Lipid accumulation and modulated activation in pDCs and CD4^-^CD8α^+^ DCs may contribute to the declining responses to vaccination and infection with age.

## Background

Aging is associated with a decline in immune function, termed immunosenescence, which results in decreased vaccine efficacy and increased incidence of infection, cancer and autoimmunity [1-4]. This decrease in immune competence has been attributed to age-related changes to the numbers and function of T- and B-lymphocytes [2]. However, the role of antigen presenting cells (APCs), in particular dendritic cells (DCs), in aging is becoming apparent. DCs are professional APCs which initiate and control immune responses against pathogenic antigens (Ag) and facilitate tolerance toward self and harmless environmental Ag [5]. In cancer and atherosclerosis, pathologies associated with increased circulating lipid concentrations, DC lipid accumulation has been associated with dysfunction [6].

Circulating lipid concentrations also significantly increase with healthy aging [7]. Thus, tissue DCs in otherwise healthy elderly hosts might be exposed to elevated lipid levels leading to DC lipid accumulation. Aging is associated with inflammaging, a low-grade, chronic, systemic inflammatory state involving increased levels of circulating pro-inflammatory cytokines such as interleukin-1 (IL-1), interleukin-6 (IL-6) and tumour necrosis factor (TNF) [8] which may contribute to the increased prevalence of autoimmunity seen in the aged [1]. Lipid uptake by DCs may lead to inappropriate DC activation such that they secrete these pro-inflammatory factors and contribute to inflammaging. Moreover, it is possible that lipid-mediated DC dysfunction might contribute to the age-related decline in immune function and that this might be an early event that precedes the development of age-related pathologies.

The exact mechanisms of lipid accumulation in DCs are poorly understood. Previous studies on macrophages have identified lipoprotein lipase (LPL) [9], scavenger receptors [10-14] and the very low density lipoprotein receptor (VLDLr) [15] as significant contributors to lipid uptake, therefore DCs may use the same receptors to take up lipids during aging. Changes to these receptors on DCs during aging had not been examined.

Until now, no studies had investigated the influence of age on DC subset distribution, their activation status, lipid content or expression of molecular mediators of lipid uptake in multiple tissues from healthy hosts. Therefore, this study aimed firstly to determine if conventional, plasmacytoid and cross-presenting DCs from apparently healthy elderly mice acquire greater lipid levels relative to younger mice despite being housed in the same environment and being on the same diet. Secondly, we aimed to identify possible mechanisms by which lipids are taken up by elderly-derived DCs. We examined DC lipid content and phenotype including molecules associated with antigen presentation and lipid uptake in secondary lymphoid organs, liver and lungs, from young adult (equivalent to 18 to 20 human years) and elderly (equivalent to 56 to 69 human years) mice [16,17].

## Results

### CD11c^+^ DC proportions increase in elderly murine tissues

**Figure 1:**
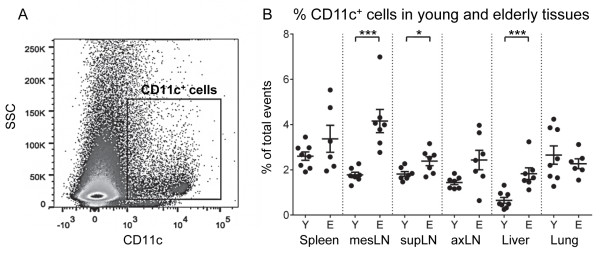
**CD11c**^**+ **^**dendritic cell proportions increase in elderly murine tissues.** Murine tissues were harvested, disaggregated into single cell suspensions and stained for CD11c^+^ DCs. CD11c^+^ DCs were identified and gated relative to the isotype control using flow cytometry **(A)**. The percentages of cells positive for CD11c in tissues are shown in young and elderly mice **(B)**. Data is shown as individual values (dots) and mean ± SEM, and is pooled from 2 separate experiments, total n = 7 – 8 mice/group. * = p ≤ 0.05; ** = p ≤ 0.01; *** = p ≤ 0.001; **** = p ≤ 0.0001.

Spleens, lymph nodes (LNs) from different sites, lungs and livers from young and elderly mice were disaggregated into single cell suspensions and stained for CD11c^+^ DCs (Figure 1A). The percentage of total cells that were CD11c^+^ DCs increased in all elderly-derived tissues (compared to young mice) except spleens and lungs, in which CD11c^+^ DC proportions did not change. Significant differences were seen in mesenteric (mes) LNs, superficial cervical (sup) LNs and livers (Figure 1B). As total body and organ weights increased with age (Additional file 1: Figure S1A and S1B), CD11c^+^ DCs were also calculated as DCs/mg of tissue; the data show that age-related changes were maintained in spleens, livers and lungs (Additional file 1: Figure S1C). Accurate weights for individual lymph nodes were not recorded therefore cell/mg could not be calculated. These data indicate that aging is associated with an increase in the percentage of DCs in most tissues except the spleen and lungs.

### CD11c^+^ DC subsets are redistributed in elderly murine tissues

**Figure 2:**
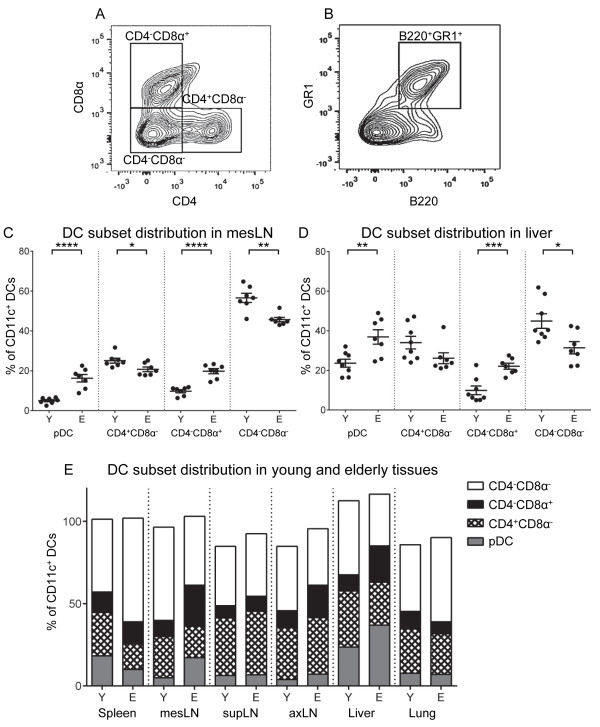
**Redistribution of CD11c**^**+ **^**DC subsets in elderly-derived mesLNs and livers.** CD4^+^CD8α^-^, CD4^-^CD8α^+^, CD4^-^CD8α^-^ DCs **(A)** and B220^+^GR1^+^ pDCs **(B)** were identified within gated CD11c^+^ cells. DC subset distribution is shown as a percentage of the CD11c^+^ cells in mesLN **(C)** and liver **(D)**; data is shown as individual values and mean ± SEM. * = p ≤ 0.05; ** = p ≤ 0.01; *** = p ≤ 0.001; **** = p ≤ 0.0001. The distribution of CD11c^+^ DC subsets in the remaining tissues are shown as mean percentages of CD11c^+^ cells **(E)**. Data in **(C)**, **(D)** and **(E)** are pooled from 2 separate experiments, total n = 7 – 8 mice/group.

The same tissues were analysed for conventional CD4^+^CD8α^-^, CD4^-^CD8α^+^, CD4^-^CD8α^-^ DC subsets (Figure 2A) and CD11c^+^B220^+^GR1^+^ pDCs (Figure 2B).

CD4^-^CD8α^-^ DCs were the dominating subpopulation in young adult mesLNs, followed by CD4^+^CD8α^-^ DCs, with CD4^-^CD8α^+^ and pDCs being rarer subpopulations (Figure 2C). Similar data were seen in supLNs and axillary (ax) LNs (Figure 2E). pDC and CD4^-^CD8α^+^ DC percentages significantly increased, whilst CD4^+^CD8α^-^ and CD4^-^CD8α^-^ DCs significantly decreased in mesLNs from elderly relative to young mice (Figure 2C). We observed increased pDC and CD4^-^CD8α^+^ DC proportions in most LNs of elderly mice, with the exception of pDCs in supLNs which did not change (Figure 2E). Furthermore, although the CD4^-^CD8α^-^ subpopulation remained dominant, pDCs and CD4^-^CD8α^+^ DCs represented a greater fraction of total DCs in elderly-derived mesLNs.

CD4^-^CD8α^-^ DCs were also the dominant DC subset in young-derived spleens, followed by CD4^+^CD8α^-^ DCs, pDCs and CD4^-^CD8α^+^ DCs. There were significant decreases in the proportions of splenic pDCs and CD4^+^CD8α^-^ DCs and a significant increase in CD4^-^CD8α^-^ DCs with aging (Figure 2E); age-related changes in pDCs and CD4^+^CD8α^-^ DCs were maintained when calculated as cells/mg of tissue (Additional file 1: Figure S1D).

Liver DC subset distribution was similar to mesLNs in young mice, with CD4^-^CD8α^-^ DCs being the numerically dominant population, followed by CD4^+^CD8α^-^ DCs. However, pDCs represented a greater proportion of CD11c^+^ DCs, and CD4^-^CD8α^+^ DCs were the rarest subset (Figure 2D). Similar to mesLNs, pDCs and CD4^-^CD8α^+^ DCs significantly increased and CD4^-^CD8α^-^ DCs significantly decreased in elderly-derived livers relative to their younger counterparts (Figure 2D). Similar trends were seen in the pDC and CD4^-^CD8α^+^ DC subsets when expressed as cells/mg, although the differences did not reach statistical significance (Additional file 1: Figure S1E). These data show that pDCs and CD4^-^CD8α^+^ DCs increased in elderly-derived livers and pDCs became the numerically dominant population.

CD4^-^CD8α^-^ DCs also dominated young adult lungs, followed by CD4^+^CD8α^-^ DCs, CD4^-^CD8α^+^ DCs and pDCs (Figure 2E). Age-related changes in elderly-derived lungs included significant increases in CD4^-^CD8α^-^ DCs, decreased CD4^-^CD8α^+^ DCs and no change in pDC proportions (Figure 2E). In contrast, no age-related changes were seen when expressed as cells/mg of tissue (data not shown).

Overall, these data show that increasing age was associated with a redistribution of DC subsets in secondary lymphoid organs as well as in liver and lungs. There was an increase in pDCs and CD4^-^CD8α^+^ DC proportions in livers and most lymph nodes. In contrast, pDC numbers did not change in lungs, and decreased in elderly-derived spleens.

### The steady state activation status of DC subsets changes with increasing age

**Figure 3:**
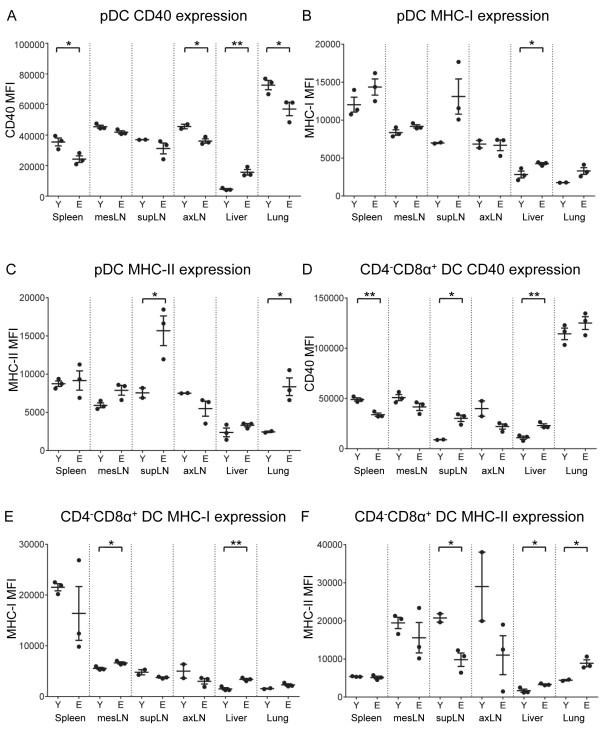
**pDC and CD4**^**-**^**CD8α**^**+ **^**DC activation status is influenced by age.** The activation status of pDCs and CD4^-^CD8α^+^ DCs was evaluated by co-expression of CD40 **(A and D)**, MHC-I **(B and E)** and MHC-II **(C and F)**. Representative data from 1 of 2 experiments is shown as individual mean fluorescent intensities (MFIs) and mean ± SEM, total n = 7 – 8 mice/group. * = p ≤ 0.05; ** = p ≤ 0.01; *** = p ≤ 0.001; **** = p ≤ 0.0001.

The aging process and/or age-related lipid accumulation may impact on the activation status of DCs. Therefore expression of surface markers associated with Ag presentation and T lymphocyte stimulation (CD40, MHC class-I and MHC class-II molecules) was examined.We first examined the pDC subset. Relative to young adult mice CD40 expression on pDCs significantly decreased with age in spleens, axLNs and lungs, yet significantly increased in livers (Figure 3A). MHC class-I expression significantly increased on pDCs in elderly-derived livers and showed an increasing trend in elderly-derived lungs, but did not change in spleens, mesLNs, supLNs and axLNs (Figure 3B). MHC class-II expression significantly increased in elderly-derived supLNs and lungs, showed an increasing trend in elderly-derived mesLNs and did not change in elderly-derived spleens, axLNs and livers (Figure 3C).

When examining CD4^+^CD8α^-^ DCs, expression of CD40 showed an increasing trend in elderly-derived axLNs, but did not change in other organs (Additional file 2: Figure S2A). MHC class-I expression on CD4^+^CD8α^-^ DCs did not change with age in any organ examined (Additional file 2: Figure S2B) and MHC class-II expression on CD4^+^CD8α^-^ DCs varied in the different tissues however, no age-related changes were seen (Additional file 2: Figure S2C).

CD40 expression significantly increased on CD4^-^CD8α^+^ DCs from elderly-derived livers and supLNs and significantly decreased on CD4^-^CD8α^+^ DCs in spleens (Figure 3D). MHC class-I expression on CD4^-^CD8α^+^ DCs significantly increased in elderly-derived mesLNs and livers and did not change in lungs, supLNs, axLNs and spleens (Figure 3E). MHC class-II expression on CD4^-^CD8α^+^ DCs displayed a significant increase in elderly-derived livers and lungs and a significant decrease in elderly supLNs (Figure 3F). No age-related changes in MHC class-II expression on CD4^-^CD8α^+^ DCs were seen in the other organs (Figure 3F).

CD40 expression on CD4^-^CD8α^-^ DCs significantly decreased in elderly-derived lungs and axLNs relative to their younger counterparts; no changes were seen in the other tissues (Additional file 2: Figure S2D). MHC class-I expression on CD4^-^CD8α^-^ DCs increased in elderly-derived spleens, decreased in supLNs and did not change in other tissues (Additional file 2: Figure S2E). Finally, MHC class-II expression on CD4^-^CD8α^-^ DCs did not change with age in any organ examined (data not shown).

Taken together, these data indicate that there are complex, organ-specific changes in DC activation status with increasing age. CD4^-^CD8α^+^ DCs displayed organ-specific CD40, MHC class-I and MHC class-II expression; e.g. CD40 expression increased in elderly-derived supLNs and livers, but decreased in spleens, whilst MHC class-I expression increased in mesLNs and livers, but did not change in other organs examined.

Age-related changes in pDC activation status were also organ-specific. CD40 expression by pDCs decreased in spleens, axLNs and lungs, but significantly increased in livers. Conversely, pDC expression of MHC class-I increased with age in liver, whilst MHC class-II expression increased with age in supLNs and lungs.

### Lipid content is influenced by age and DC subset

Until now, no studies had examined lipid accumulation by tissue DC subsets in aging. Different sites such as the mesLN and liver are exposed to higher levels of triglyceride rich lipoproteins due to their proximity to the gut and role in lipid metabolism respectively. Therefore, the influence of anatomical location on neutral lipid content in DC subsets in the aging context was examined using BODIPY, a lipophilic fluorescent stain.

**Figure 4:**
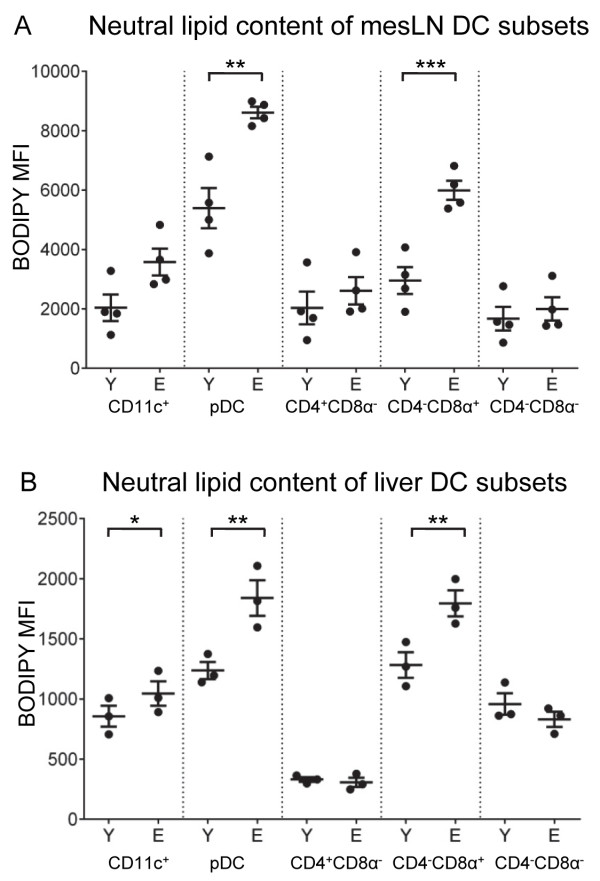
**Neutral lipid content is influenced by age and DC subpopulation in mesLNs and livers.** The lipid content of DC subpopulations from murine mesLNs **(A)** and livers **(B)** was evaluated by flow cytometry following BODIPY staining. Representative data from 1 of 2 experiments is shown as individual MFIs and mean ± SEM, total n = 7 – 8 mice/group. Significance was calculated using two-way ANOVA. * = p ≤ 0.05; ** = p ≤ 0.01; *** = p ≤ 0.001; **** = p ≤ 0.0001.

Our data show that regardless of age pDCs in all anatomical locations contained the most lipid, followed by CD4^-^CD8α^+^ DCs, and that lipid content in these DCs increased with age. For example, in young-derived mesLNs, pDCs contained the most lipid, followed by CD4^-^CD8α^+^ DCs, CD4^+^CD8α^-^ DCs and CD4^-^CD8α^-^ DCs; lipid content increased with age in the total CD11c^+^ DC population which was likely due to the significant increases in pDC and CD4^-^CD8α^+^ DC lipid content (Figure 4A). Similar age-related increases in DC lipid content were seen in (i) supLNs (Additional file 3: Figure S3A), with significant increases in lipid content of pDCs and CD4^-^CD8α^+^ DCs with age; (ii) axLNs in which elderly-derived pDC lipid content significantly increased (Additional file 3: Figure S3B); and (iii) livers, again with significant increase in CD11c^+^ DC lipid content with age, correlating to increases in pDC and CD4^-^CD8α^+^ DC lipid content (Figure 4B). There were no significant age-related changes in lipid content in the remaining CD4^+^CD8α^-^ and CD4^-^CD8α^-^ subsets.

Spleens and lungs were different as whilst pDCs contained high lipid levels, followed by CD4^-^CD8α^+^ DCs in young-derived spleens, no significant age-related changes in lipid levels were seen (data not shown). These data show that pDCs and cross-presenting CD4^-^CD8α^+^ DCs contain the most lipids in all young murine tissues examined. Furthermore, the lipid content of both these subsets increased with age in livers and LNs, which may suggest increased lipid uptake.

### Scavenger receptor, LPL and VLDLr expression is influenced by age and DC subset

We next asked how lipids might be taken up by DCs. Lipoprotein specific receptors, such as LDLr [18] and VLDLr [15], LPL [9] and the scavenger receptors low density lipoprotein receptor-related protein 1 (LRP-1) [10,14], CD36 [12] and CD68 [13] mediate the uptake of modified and native lipoproteins by macrophages and may also play a role in DC lipid accumulation [6]. Therefore, we determined if DCs from different anatomical locations differentially express LPL, LRP-1, CD36, CD68 and VLDLr, and whether age influences their expression.

**Figure 5:**
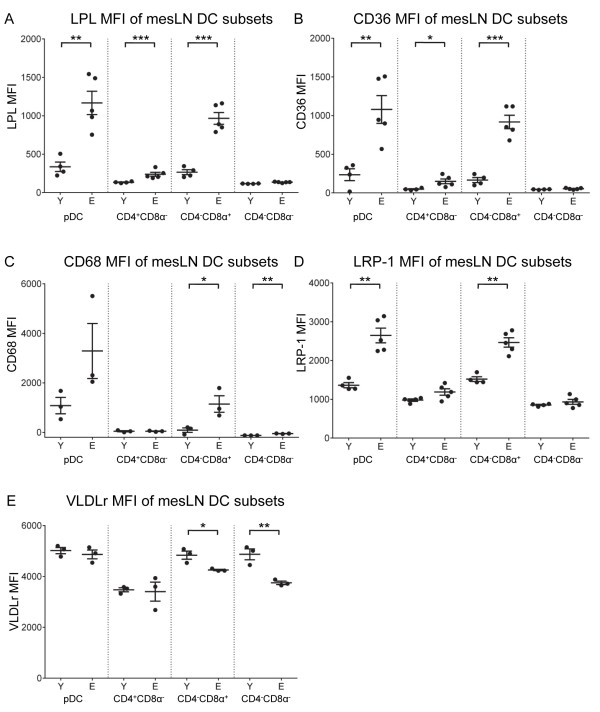
**LPL, scavenger receptor and VLDLr expression is influenced by age in mesLNs.** LPL **(A)**, CD36 **(B)**, CD68 **(C)**, LRP-1 **(D)** and VLDLr **(E)** expression on DC subsets was assessed by flow cytometry. Representative data from 1 of 2 experiments is shown as individual MFIs and mean ± SEM, total n = 7 – 8 mice/group. * = p ≤ 0.05; ** = p ≤ 0.01; *** = p ≤ 0.001; **** = p ≤ 0.0001.

LPL, scavenger receptor and VLDLr expression levels (measured using MFI) were low in all DC subsets in young-derived mesLNs (Figures 5A to 5E). With aging, LPL (Figure 5A) and CD36 expression (Figure 5B) in mesLNs significantly increased in pDCs, CD4^+^CD8α^-^ and CD4^-^CD8α^+^ DCs; no changes were seen in CD4^-^CD8α^-^ DCs. In elderly-derived mesLNs, CD68 expression significantly increased in CD4^-^CD8α^+^ and CD4^-^CD8α^-^ DCs, with a similar trend in pDCs, but no changes in CD4^+^CD8α^-^ DCs (Figure 5C). LRP-1 expression also significantly increased in pDCs and CD4^-^CD8α^+^ DCs but not in other DC subsets (Figure 5D). In contrast, VLDLr expression significantly decreased in CD4^-^CD8α^+^ and CD4^-^CD8α^-^ DCs and did not change in the remaining DC subsets (Figure 5E).

Age -related increases in levels of LPL by pDCs and CD4^-^CD8α^+^ DCs in other lymph nodes were not significant, however there were age-related increases for CD36 in pDCs and CD4^-^CD8α^+^ DCs, and similarly for CD68 in supLN pDCs and axLN CD4^-^CD8α^+^ DCs (data not shown).

LPL expression in elderly-derived spleens significantly increased in pDCs, CD4^-^CD8α^+^ and CD4^-^CD8α^-^ DCs (Additional file 4: Figure S4A). CD36 expression significantly increased in CD4^+^CD8α^-^ DCs, and did not change in the other subsets (Additional file 4: Figure S4B). CD68 expression significantly increased on pDCs and CD4^-^CD8α^+^ DCs (Additional file 4: Figure S4C). LRP-1 (Additional file 4: Figure S4D) and VLDLr (Additional file 4: Figure S4E) expression significantly decreased in all DC subpopulations in elderly-derived spleens.

**Figure 6:**
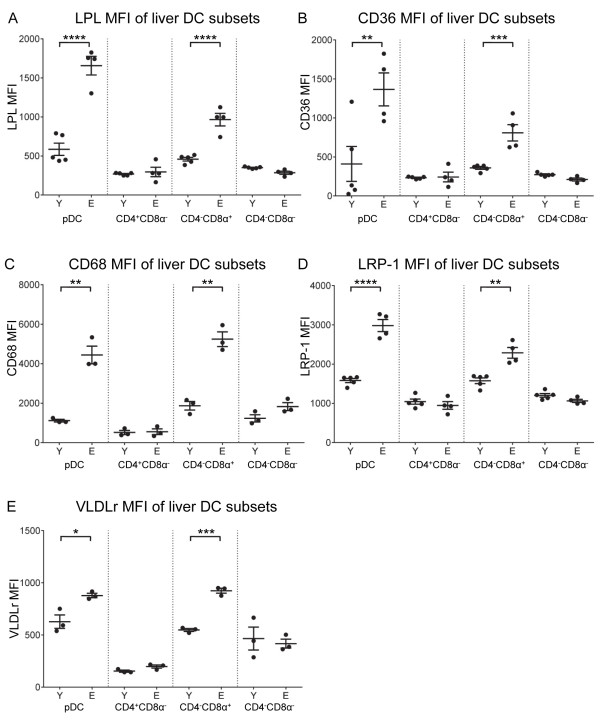
**LPL, scavenger receptor and VLDLr expression is influenced by age and DC subset in livers.** LPL **(A)**, CD36 **(B)**, CD68 **(C)**, LRP-1 **(D)** and VLDLr **(E)** expression on liver DC subsets was assessed by flow cytometry. Representative data from 1 of 2 experiments is shown as individual MFIs and mean ± SEM, total n = 7 – 8 mice/group. * = p ≤ 0.05; ** = p ≤ 0.01; *** = p ≤ 0.001; **** = p ≤ 0.0001.

Expression levels of LPL (Figure 6A), CD36 (Figure 6B), CD68 (Figure 6C), LRP-1 (Figure 6D) and VLDLr (Figure 6E) significantly increased in pDCs and CD4^-^CD8α^+^ DCs in elderly-derived livers; no changes in expression levels of these receptors were seen in the remaining DC subsets.

In elderly-derived lungs, LPL (Additional file 5: Figure S5A), LRP-1 (Additional file 5: Figure S5B) and CD68 (Additional file 5: Figure S5C) expression significantly increased on pDCs and CD4^-^CD8α^+^ DCs. Conversely, whilst CD36 expression significantly decreased on CD4^+^CD8α^-^ and CD4^-^CD8α^-^ DCs, it did not change on pDCs or CD4^-^CD8α^+^ DCs (Additional file 5: Figure S5D), and VLDLr expression in the latter significantly increased with age (Additional file 5: Figure S5E).

Overall, the data show that tissue pDCs and CD4^-^CD8α^+^ DCs acquire lipids in numerous tissues during aging, which is associated with increased LPL and scavenger receptor expression, particularly in livers and mesLNs.

## Discussion

There is limited evidence that DC biology may be altered with aging. Furthermore studies on age-related changes to DC numbers and activation status are conflicting. This is particularly true for human studies of circulating DC subsets; some have shown a significant decrease in circulating pDC numbers with no change in myeloid DC numbers [19-22], whilst others reported a decrease in myeloid DC populations with no change in pDC numbers [23]. Similarly, whilst several studies have shown that expression of key surface molecules related to antigen presentation do not vary due to age [24-26], others have described a significant drop in HLA-DR expression on elderly-derived DCs [27]. Several factors are known to influence DC function, and recently the influence of lipids and lipid accumulation on DC biology and function in both healthy and pathological contexts has become apparent. However, to our knowledge, no studies have examined DC subset distribution, activation status, or lipid accumulation in the context of healthy aging.

Our in vivo studies using mice show that DC subset tissue distribution is altered with aging. We observed an increase in the percentages of CD11c^+^ DCs in all tissues except the lungs. Interestingly, this increase was not universal across all DC subsets, but was attributable to significant increases in pDCs and CD4^-^CD8α^+^ DCs in most tissues. Plasmacytoid DCs and cross-presenting CD4^-^CD8α^+^ DCs play key roles in anti-viral and anti-tumour immunity [28]. Cross-presenting DCs are the only DCs that can induce CD8^+^ T-cell mediated immune responses following vaccination, viral challenge or encounter with pathogenic self Ag. Furthermore, it has recently been shown that in non-pathological conditions pDCs and CD4^-^CD8α^+^ DCs may also play a role in immune tolerance [28,29]. Previous studies have reported that pDC numbers in human blood decrease with age [22,30]. Taken with data from this study, we postulate that there is an age-related redistribution of DC subsets, and that pDCs may accumulate in secondary lymphoid tissues and livers with increasing age leaving lower numbers available for circulation. Alterations in DC tissue distribution may also be due to the age-related decreases in T- and B- lymphocytes, which have been shown to result in a decreased efficiency of immune responses [2]. Thus, we postulate that as modulators of immune responses, DCs in the elderly migrate to T- and B-cell rich sites such as the LNs in an attempt to restore immune function through increased lymphocyte stimulation.

Data from this study also suggests that DC activation status in the absence of obvious pathology is influenced by age. Changes in activation status of pDCs were organ-specific, indicating that the tissue microenvironment is a major influencing factor in DC biology. Expression of CD40 was decreased in spleens, axLNs and lungs, suggesting a decreased capacity for T-cell licencing via CD40 in these organs. However, T-cell licencing capacity may be increased in elderly livers, due to the observed increase in CD40 expression with age. There was also an age-related increase in expression of MHC class-I and class-II molecules, suggesting an increased capacity for Ag presentation. Furthermore, activated pDCs are the primary producer of type I interferons, which drive the production of other pro-inflammatory cytokines, and the activation and recruitment of leukocytes [31]. Changes in activation status of conventional DC subsets were also organ-specific. For example, CD40 expression by CD4^-^CD8α^+^ DCs increased in elderly-derived supLNs and livers, but decreased in spleens. Changes in MHC class-I and class-II expression also showed organ-specific patterns. Although the exact phenotypic modifications were organ-specific, the result was an increased steady-state activation status of CD4^-^CD8α^+^ DCs in non-lymphoid organs. Activated CD4^-^CD8α^+^ DCs produce large quantities of the pro-inflammatory cytokine IL-12, which similarly drives lymphocyte activation and recruitment during immune responses [32]. Whilst our data suggests that elderly DCs may have improved antigen presentation capacity (via increased expression of MHC molecules) in some tissue sites, this contrasts with evidence that T cell responses are decreased in elderly hosts [33,34]. This may be explained by intrinsic defects in elderly T cells which may render them unable to respond to stimulation by DCs [33,34]. Reduced expression of co-stimulatory molecules on elderly DCs, such as the reduction in CD40 expression that we observed in some tissue sites, may also limit T cell responses in elderly hosts. Furthermore, increased expression of inhibitory molecules, such as PD-L1, on elderly DCs (our unpublished data and [35]) may also contribute to reduced T cell responses in the elderly. Thus, the outcomes of age-related changes in DC activation status are complex and may depend on other factors, such as the tissue microenvironment and age-related changes in other immune cells.

Ibrahim et al. demonstrated that the lipid content of hepatic DCs was a determinant of their immunogenicity, and that lipid-laden DCs produced higher levels of TNF-α [36]. TNF-α is a powerful pro-inflammatory cytokine which is associated with numerous pathologies [37,38]. Thus, increases in pDC and CD4^-^CD8α^+^ DC activation may result in increased secretion of pro-inflammatory factors and the establishment of a pro-inflammatory microenvironment. Polarizing DCs toward a pro-inflammatory phenotype may alter the types of immune responses generated at different organ sites. For example, mesLNs and livers are important sites for the development of oral tolerance [39], which relies on Ag presentation by pDCs [40]. We observed increased lipid levels and activation of liver pDCs, which may suggest increased immunogenicity, as observed by others [36], possibly resulting in a breakdown of tolerance in these sites.

The establishment of an age-associated pro-inflammatory microenvironment may contribute to chronic low-grade activation of DCs, which could promote and/or facilitate the onset of pathologies associated with aging such as autoimmunity or cancer [8]. Furthermore, the development of a semi-mature phenotype by pDCs and CD4^-^CD8α^+^ DCs may inhibit the development of adaptive immune responses against viral infections and tumours, thus contributing to impaired clearance of viral infections, a reduced efficiency of vaccinations, and increased incidence of cancer in the elderly. Furthermore, studies of anti-cancer immunity have shown that in the presence of tumour cells and tumour-derived factors, DCs readily accumulate lipids resulting in a decreased ability to stimulate T-cell immune responses [6].

Despite the associations seen between lipid content and altered function, no studies have yet established how DCs accumulate lipid. Lipid accumulation in macrophages has been well studied and numerous receptors and lipid transporters have been identified as significant contributors including lipoprotein specific receptors (LDLr and VLDLr), scavenger receptors (Msr-1, CD36, CD68) and LPL [6,9-14,36,41]. The latter is a serine hydrolase that releases free fatty acids from circulating triglyceride-rich lipoproteins, but it can also facilitate uptake of triglyceride-rich lipoproteins by lipoprotein receptors, including LRP-1 [42] and VLDLr [43]. There are few studies on DCs, but Herber et al. [6] found a role for Msr-1 (CD204), and Ibrahim et al., who studied liver DCs, found a role for Msr-1, CD68 and LPL [36]. In our study, the increased lipid content of DCs from aged mice was consistently accompanied by increased LPL. A number of receptors can work in concert with LPL to cause lipid accumulation, e.g. by mediating uptake of the fatty acids released by LPL (e.g. CD36) or by increased lipoprotein uptake through formation of a complex with lipoprotein receptors (e.g. LRP-1 or VLDLr); in our study, liver DCs of aged mice had increases in both CD36 and LRP-1, as well as VLDLr and CD68. In other tissues, the increased DC lipid and LPL expression in pDCs and CD4^-^CD8α^+^ DCs was also associated with an increased expression of receptors, but the type and number of receptors involved varied. Lung and mesLN-derived DCs showed upregulation of some but not all scavenger receptors, whereas for spleen, whilst CD68 was increased, LRP-1 and VLDLr expression decreased.

The elevation in DC lipid levels may be explained by an increase in LPL, the lipoprotein receptors or a combination of both. The fact that LPL was so consistently elevated with age suggests an important role for this particular enzyme. Moreover, our observation that different receptors may be involved at different tissue sites is not surprising since DCs in these different sites may be exposed to different milieu. For example, the liver plays a major role in lipid metabolism, and is a major site of lipoprotein synthesis, resulting in exposure of DCs to higher concentrations of lipoproteins, particularly triglyceride-rich lipoproteins. Intestinally derived triglyceride-rich lipoproteins reach circulation via mesenteric lymph and the thoracic duct; as such mesLNs may also be exposed to high concentrations of these lipoproteins, and of the lymph nodes examined, mesLNs showed more marked age-related changes in lipid accumulation, LPL and expression of receptors potentially involved in lipid uptake. Lipid levels including plasma cholesterol and triglycerides increase with age in humans and animal models [44-46]. However, whilst exposure to lipids may result in changes in DC phenotype and function, other environment-derived factors, particularly in the context of aging, also likely modulate the expression of these molecules. Such factors may include age-related increases in modified lipoprotein particles such as oxidised lipoproteins which have been shown to stimulate the increased expression of scavenger receptors in macrophages [47]. Interferon gamma has been shown to increase uptake of triglyceride-rich lipoproteins in macrophages via an LPL dependent mechanism [41], and an age dependent inflammatory process may thus have similar effects on DCs.

## Conclusions

Aging is accompanied by increases in tissue pDCs and CD4^-^CD8α^+^ DCs in association with increasing DC lipid accumulation and partial activation. Interestingly, these effects were most distinct in tissues which may be exposed to elevated levels of lipoproteins such as the mesLNs and liver, suggesting that the immunomodulatory effects of lipids are most at play in these locations. Increased activation of pDCs and CD4^-^CD8α^+^ DCs has been associated with the increased production of pro-inflammatory factors such as type-I interferons, which may contribute to the establishment of a pro-inflammatory microenvironment. Such pro-inflammatory environments have been implicated in promoting the development and maintenance of cancer and autoimmunity, pathologies common in elderly hosts. Furthermore lipid-mediated DC dysfunction, characterised by a decreased capacity to initiate T-cell mediated immune responses, may also result in a decreased capacity to clear viral pathogens, resulting in an increased prevalence of infection and decreased efficiency of vaccinations in the elderly.

## Methods

### Mice

Young adult (6–8 weeks) and elderly (22–24 months) female C57BL/6J mice obtained from the Animal Resources Centre (Perth, Western Australia) were housed under pathogen-free conditions at the Curtin University animal facility. Animal experiments were performed as per Curtin University Animal Ethics Committee (AEC) approval number AEC-2012-21.

### Murine tissue collection and processing

Secondary lymphoid organs (spleens, mesenteric lymph nodes, superficial cervical lymph nodes and axillary lymph nodes) were processed into single cell suspensions between two frosted glass slides. Livers and lungs were sliced into 0.5 mm^3^ sections then digested for 90 mins at 37°C in type 4 collagenase (1 mg/mL; Sigma-Aldrich, Missouri, USA) and bovine pancreatic DNase (0.1 mg/mL, Sigma-Aldrich) in FACS buffer consisting of PBS with 2% fetal calf serum (FCS; ThermoScientific, Massachusetts, USA) and 1% bovine serum albumin (BSA; Sigma-Aldrich). Samples were then triturated for 5 mins, filtered and incubated at 4°C for 10 mins in FACS buffer containing 20% FCS to aid cell recovery.

### Flow cytometry: phenotyping, lipid quantification and receptor expression

Murine DCs were surface stained for 30 mins in the dark at 4°C with fluorescently labelled antibodies against CD11c (clone N418, BD), CD4 (clone RM4-5, BD), CD8α (clone 53–6.7, BD), GR1 (clone RB6.8C5, BD), B220 (clone RA3.6B2, BD), CD40 (Biolegend, California, USA), MHC class-I (Biolegend) and MHC class-II (Biolegend). For intracellular lipid quantification, cells were incubated for 15 mins at 4°C in the dark with BODIPY 493/503 (Invitrogen).

For staining molecules associated with lipid uptake, cells were fixed in 1% paraformaldehyde (Sigma-Aldrich) and permeabilised with 0.1% saponin (Sigma-Aldrich). Cells were incubated with fluorescently labelled anti-CD36 (Biolegend), anti-CD68 (Biolegend), LRP-1 (Santa Cruz, California, USA), LPL (Abcam, Cambridge, UK) and VLDLr (Abcam) in the dark for 30 mins at 4°C. Before staining, mouse IgG_1_ antibodies (LPL and VLDLr) were directly conjugated to zenon reagents (Invitrogen) as per manufacturer’s instructions. Following LRP-1 staining, cells were sequentially incubated with anti-goat biotin (Dako, California, USA) and streptavidin-V500 (BD) in the dark for 30 mins at 4°C.

Following staining, samples were washed twice and resuspended in FACS buffer for acquisition on a FACSCanto II (BD) followed by analysis using FlowJo V10 software (Treestar, Oregon, USA). Isotype-matched antibodies were used as controls.

### Data analysis

Statistical significance was calculated using non-parametric Mann–Whitney Student’s t-test, using GraphPad PRISM 5 (GraphPad Software Inc, California, USA). P-values < 0.05 were considered statistically significant.

## Competing interests

The authors declare that they have no competing interests.

## Authors’ contributions

JKG: Performed the experiments, participated in data collection and analysis and helped draft the manuscript. TM: Performed the experiments, analysed the data and drafted the manuscript. CM: Contributed to design of the study and drafting the manuscript. DED: Provided the mice and some reagents. CJ: Participated in data collection and analysis, design of the study and drafting the manuscript. DJN: Designed the study, analysed the data and drafted the manuscript. All authors read and approved the final manuscript.

## Supplementary Material

Additional file 1: Figure S1 Aging is associated with increased body and organ weight and altered DC tissue distribution. Body **(A)** and organ weight of young- and elderly-derived spleens, livers and lungs **(B**). DCs were identified as per Figure 1 and expressed as CD11c^+^ cells/mg of tissue **(C)**; data shown as mean values ± SEM. DC subset distribution is shown as cells/mg of tissue in spleens **(D)** and liver **(E)**; data shown as individual values and mean ± SEM. All data is pooled from 2 separate experiments, total n = 7 – 8 mice/group. * p ≤ 0.05; ** = p ≤ 0.01; *** = p ≤ 0.001; **** = p ≤ 0.0001. Click here for file

Additional file 2: Figure S2 DC activation status is influenced by age. The activation status of CD4^+^CD8α^-^ DCs was evaluated by co-expression of CD40 **(A)**, MHC-I **(B)** and MHC-II **(C)**. Expression of CD40 **(D)** and MHC-I **(E)** was also examined on CD4^-^CD8α^-^ DCs. Representative data from 1 of 2 experiments is shown as individual mean fluorescent intensities (MFIs) and mean ± SEM (total n = 7–8 mice/group). * = p ≤ 0.05; ** = p ≤ 0.01; *** = p ≤ 0.001; **** = p ≤ 0.0001. Click here for file

Additional file 3: Figure S3 Lipid content is influenced by age and anatomical location. The lipid content of DC subsets from supLNs **(A)** and axLNs **(B)** was evaluated by flow cytometry following BODIPY staining. Representative data from 1 of 2 experiments is shown as individual MFIs and mean ± SEM (total n = 7 – 8 mice/group). * = p ≤ 0.05; ** = p ≤ 0.01; *** = p ≤ 0.001; **** = p ≤ 0.0001. Click here for file

Additional file 4: Figure S4 LPL, scavenger receptor and VLDLr expression on DC subsets is influenced by age in spleens. LPL **(A)**, CD36 **(B)**, CD68 **(C)**, LRP-1 **(D)** and VLDLr **(E)** expression on DC subsets was assessed by flow cytometry. Representative data from 1 of 2 experiments is shown as individual MFIs and mean ± SEM (total n = 7–8 mice/group). * = p ≤ 0.05; ** = p ≤ 0.01; *** = p ≤ 0.001; **** = p ≤ 0.0001. Click here for file

Additional file 5: Figure S5 LPL, scavenger receptor and VLDLr expression on DC subsets is influenced by age in lungs. LPL **(A)**, LRP-1 **(B)**, CD68 **(C)**, CD36 **(D**) and VLDLr **(E)** expression on DC subsets was assessed by flow cytometry. Representative data from 1 of 2 experiments is shown as individual MFIs and mean ± SEM (total n = 7–8 mice/group). * = p ≤ 0.05; ** = p ≤ 0.01; *** = p ≤ 0.001; **** = p ≤ 0.0001. Click here for file
