# Supplementary figures and images for: Lipid-laden partially-activated plasmacytoid and CD4−CD8α+ dendritic cells accumulate in tissues in elderly mice
Source: Immun Ageing. 2014 Jul 29;11:11. doi: 10.1186/1742-4933-11-11 (PMC4118209; doi:10.1186/1742-4933-11-11)

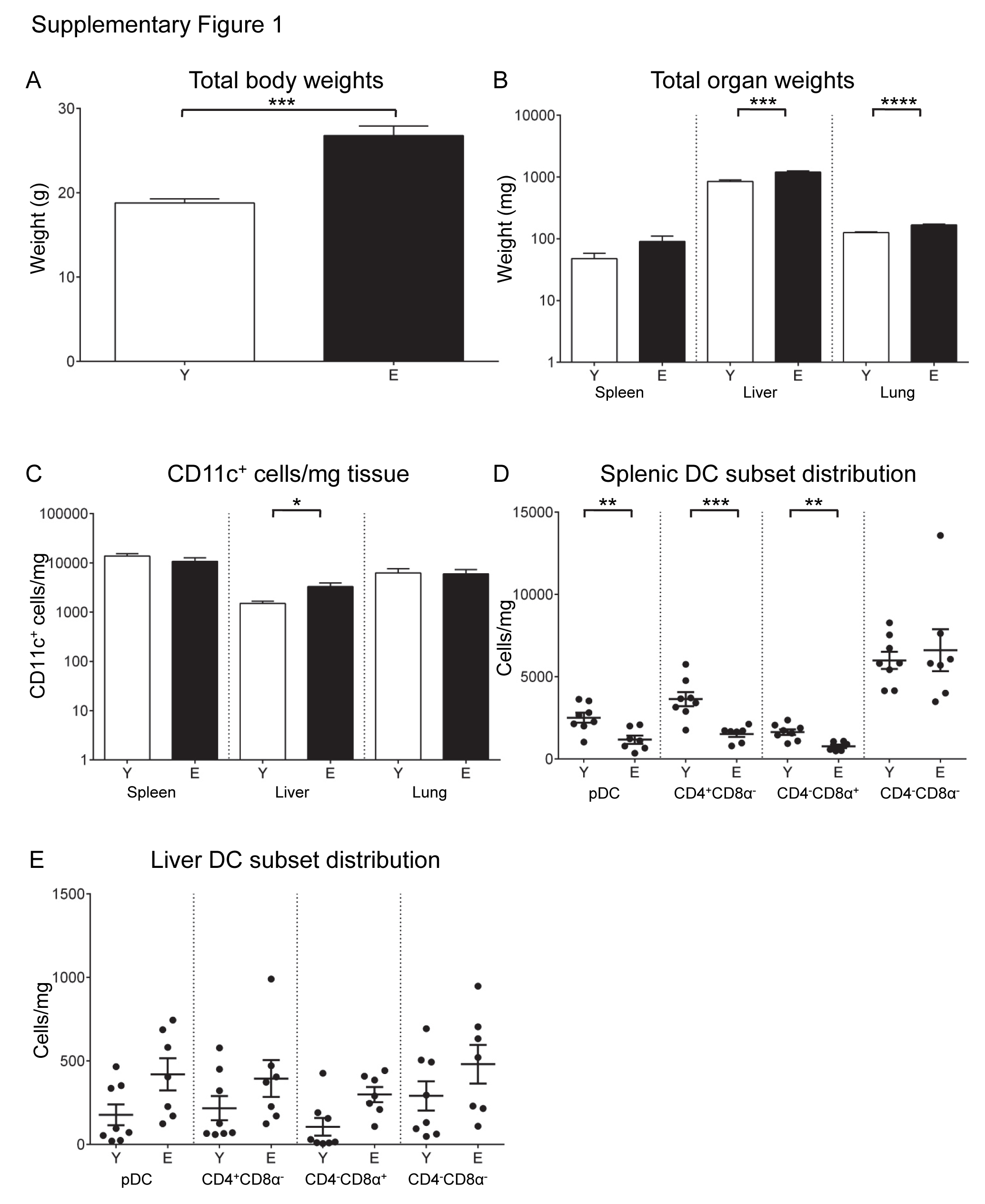

Supplement: Additional file 1: Figure S1 — Aging is associated with increased body and organ weight and altered DC tissue distribution. Body (A) and organ weight of young- and elderly-derived spleens, livers and lungs (B). DCs were identified as per Figure 1 and expressed as CD11c+ cells/mg of tissue (C); data shown as mean values ± SEM. DC subset distribution is shown as cells/mg of tissue in spleens (D) and liver (E); data shown as individual values and mean ± SEM. All data is pooled from 2 separate experiments, total n = 7 – 8 mice/group. * p ≤ 0.05; ** = p ≤ 0.01; *** = p ≤ 0.001; **** = p ≤ 0.0001. [file 1742-4933-11-11-S1.tiff]

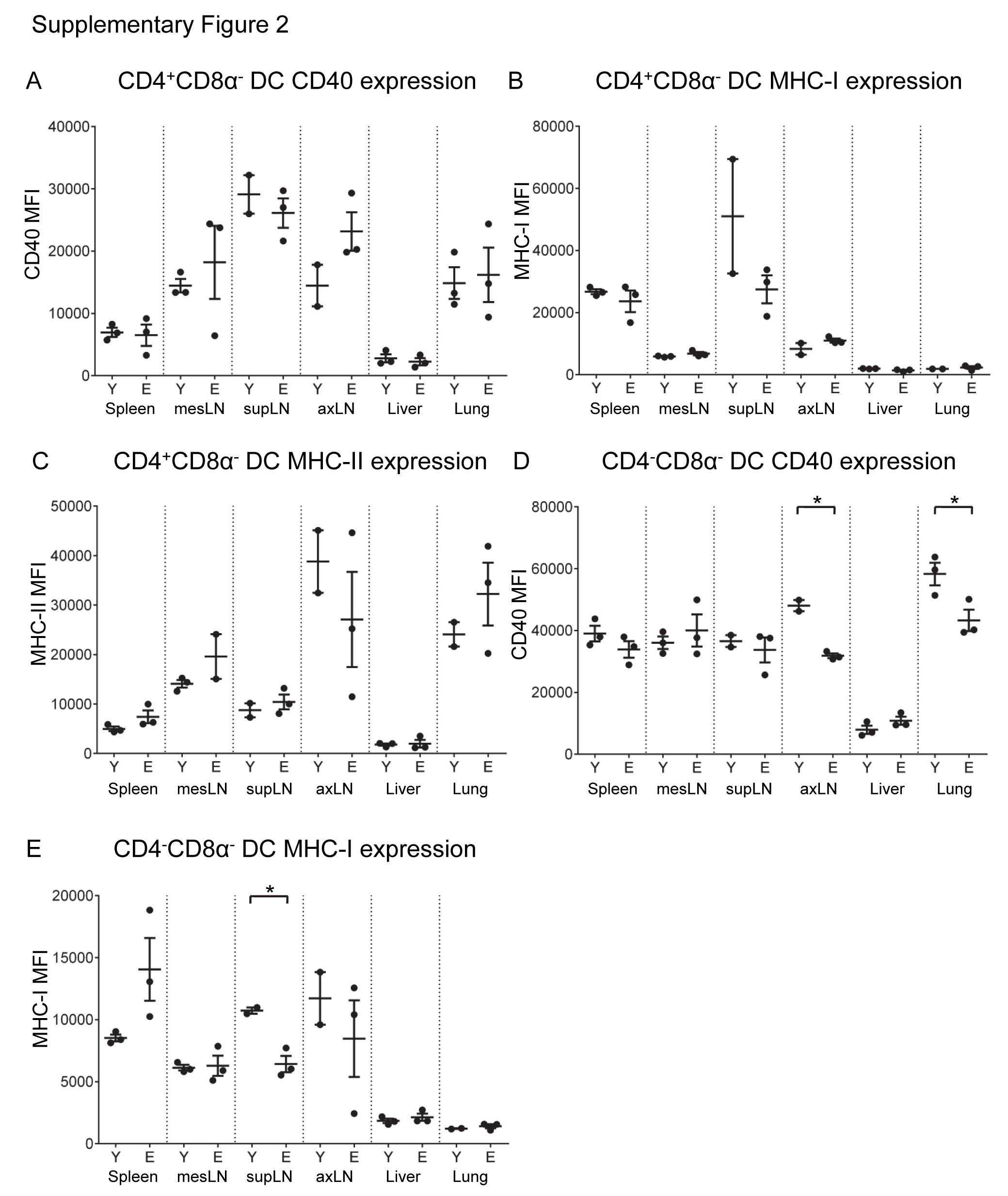

Supplement: Additional file 2: Figure S2 — DC activation status is influenced by age. The activation status of CD4+CD8α- DCs was evaluated by co-expression of CD40 (A), MHC-I (B) and MHC-II (C). Expression of CD40 (D) and MHC-I (E) was also examined on CD4-CD8α- DCs. Representative data from 1 of 2 experiments is shown as individual mean fluorescent intensities (MFIs) and mean ± SEM (total n = 7–8 mice/group). * = p ≤ 0.05; ** = p ≤ 0.01; *** = p ≤ 0.001; **** = p ≤ 0.0001. [file 1742-4933-11-11-S2.tiff]

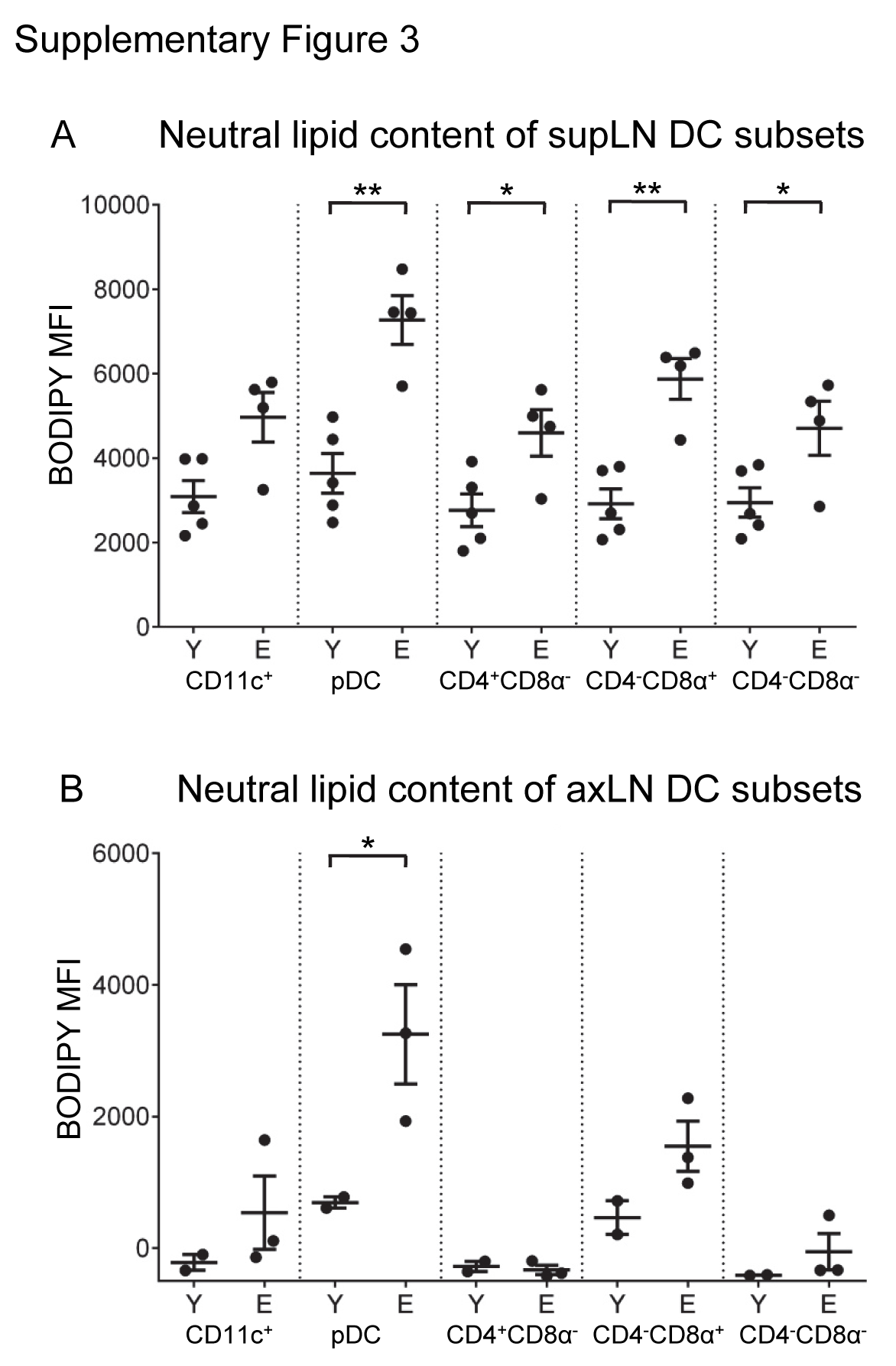

Supplement: Additional file 3: Figure S3 — Lipid content is influenced by age and anatomical location. The lipid content of DC subsets from supLNs (A) and axLNs (B) was evaluated by flow cytometry following BODIPY staining. Representative data from 1 of 2 experiments is shown as individual MFIs and mean ± SEM (total n = 7 – 8 mice/group). * = p ≤ 0.05; ** = p ≤ 0.01; *** = p ≤ 0.001; **** = p ≤ 0.0001. [file 1742-4933-11-11-S3.tiff]

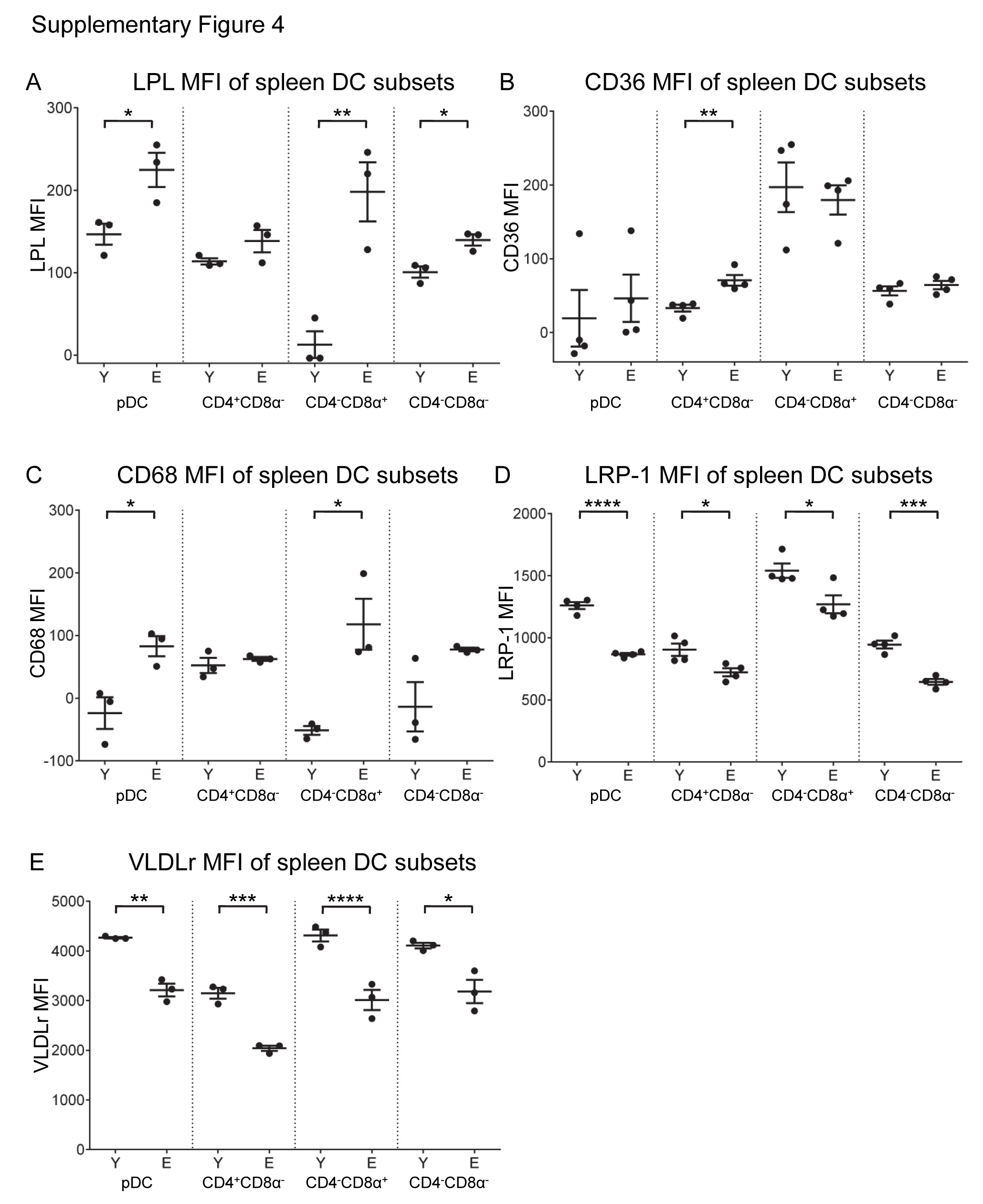

Supplement: Additional file 4: Figure S4 — LPL, scavenger receptor and VLDLr expression on DC subsets is influenced by age in spleens. LPL (A), CD36 (B), CD68 (C), LRP-1 (D) and VLDLr (E) expression on DC subsets was assessed by flow cytometry. Representative data from 1 of 2 experiments is shown as individual MFIs and mean ± SEM (total n = 7–8 mice/group). * = p ≤ 0.05; ** = p ≤ 0.01; *** = p ≤ 0.001; **** = p ≤ 0.0001. [file 1742-4933-11-11-S4.tiff]

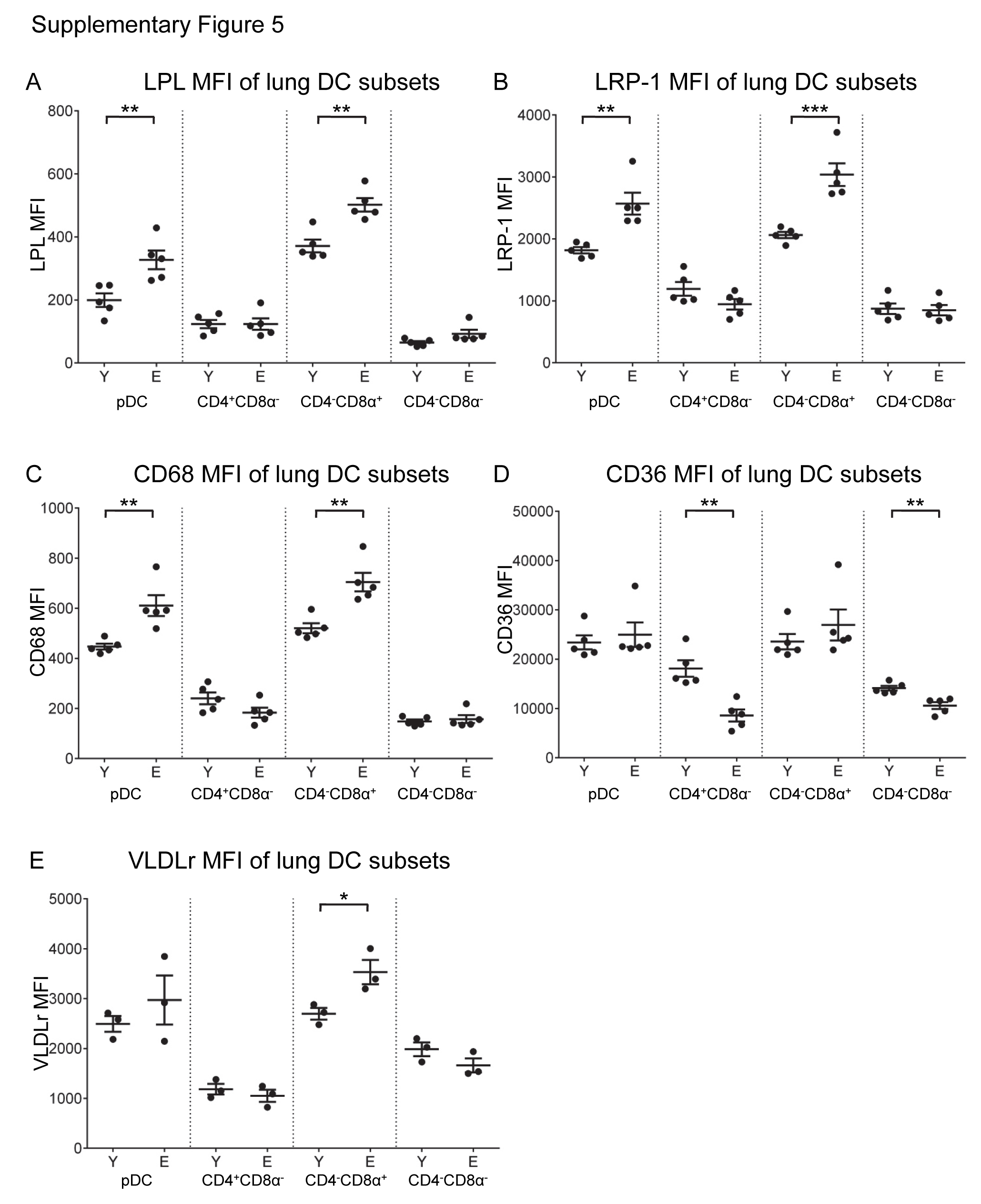

Supplement: Additional file 5: Figure S5 — LPL, scavenger receptor and VLDLr expression on DC subsets is influenced by age in lungs. LPL (A), LRP-1 (B), CD68 (C), CD36 (D) and VLDLr (E) expression on DC subsets was assessed by flow cytometry. Representative data from 1 of 2 experiments is shown as individual MFIs and mean ± SEM (total n = 7–8 mice/group). * = p ≤ 0.05; ** = p ≤ 0.01; *** = p ≤ 0.001; **** = p ≤ 0.0001. [file 1742-4933-11-11-S5.tiff]
